# The Evolution of Modeling Approaches: From Statistical Models to Deep Learning for Locust and Grasshopper Forecasting

**DOI:** 10.3390/insects17020182

**Published:** 2026-02-08

**Authors:** Wei Sui, Jing Wang, Dan Miao, Yijie Jiang, Guojun Liu, Shujian Yang, Wei You, Zhi Li, Xiaojing Wu, Hu Meng

**Affiliations:** 1 Inner Mongolia Key Laboratory of Life Health and Bioinformatics, College of Life Science and Technology, Inner Mongolia University of Science and Technology, Baotou 014010, China; gjliu@imust.edu.cn; 2 Baotou Garden Landscape Research Institute, Baotou 014010, China; sylviaju@foxmail.com (W.S.);; 3 School of Soil and Water Conservation, Beijing Forestry University, Beijing 100083, China; 4 Baotou Landscaping and Greening Development Center, Baotou 014010, China

**Keywords:** locust plague, outbreak prediction, spatiotemporal modeling, machine learning, MaxEnt, GRU, XAI

## Abstract

Locust outbreaks cause a significant threat to global food security and ecosystem stability, with particularly severe consequences in grassland regions, where grasshoppers also exert considerable ecological pressure. In comparison to grasshoppers, locusts typically occur at much larger spatial scales, as their strong migratory ability and collective movement behavior lead to greater spatial connectivity and autocorrelation. The forecasting of both locust and grasshopper outbreaks remains a formidable scientific challenge, primarily due to the complex, nonlinear spatiotemporal interactions among environmental drivers such as weather, vegetation, and soil conditions. This review compares the evolution of prediction methodologies for locust and grasshopper outbreaks, focusing on the application of deep learning (DL) methods to ecological forecasting tasks. It traces the development from traditional statistical models to classical machine learning, and ultimately to DL, assessing the strengths and limitations of key DL architectures—including Deep Neural Networks (DNNs), Convolutional Neural Networks (CNNs), Recurrent Neural Networks (RNNs), Long Short-Term Memory (LSTM) networks, and Gated Recurrent Units (GRUs)—in modeling the intricate dynamics of locust populations. While most studies have concentrated on locust outbreaks, this review emphasizes the adaptation of these models to grassland ecosystems, such as those in Inner Mongolia, where grasshopper outbreaks exhibit similarities to locust plagues but have been largely overlooked in DL research. Despite the potential of DL, challenges such as data scarcity, limited model generalizability across regions, and the “black box” issue of low interpretability remain. To address these issues, we propose future research directions that integrate Explainable AI (XAI), transfer learning, and generative models like GANs to development more robust, transparent, and ecologically grounded forecasting tools. By promoting the use of efficient architectures like GRUs within customized frameworks, this review aims to guide the development of effective early warning systems for sustainable locust management in vulnerable grassland ecosystems.

## Simple Summary

Locust outbreaks, driven by factors such as weather, vegetation, and soil conditions, pose a significant threat to agriculture and ecosystems. Since grasshoppers in grassland ecosystems exhibit similar behaviors to locusts, we adapt locust prediction models to forecast grasshopper outbreaks. This study reviews various prediction methods, including traditional statistical models, machine learning (ML), and deep learning (DL), highlighting the advantages of DL in ecological forecasting tasks as demonstrated by the existing literature. While Gated Recurrent Units (GRUs) are promising, especially in data-limited regions, challenges such as data scarcity, the specific limitations of grassland ecosystems, and model interpretability remain. This study suggests combining GRUs with advanced technologies to improve the accuracy and transparency of locust and grasshopper predictions, enhancing pest management in grassland regions.

## 1. Introduction

### 1.1. The Global Socio-Ecological Impact of Locust/Grasshopper Outbreaks

Locust infestations are widely considered one of the most destructive biological disasters, with severe consequences for global agriculture and ecological balance [1,2]. These large-scale outbreaks are characterized by their sudden onset and ability to spread across borders, continuously threatening food security, social stability, and overall ecosystem health [3,4,5,6]. Among these species, the desert locust (*Schistocerca gregaria*) is particularly notorious. Listed by the Food and Agriculture Organization (FAO) of the United Nations as one of the world’s most damaging pests, its capacity for rapid reproduction, long-distance migration, and widespread crop destruction makes it especially dangerous [1]. Other species, including the migratory locust (*Locusta migratoria*), the Moroccan locust (*Dociostaurus maroccanus*), and the Italian locust (*Calliptamus italicus*), also cause substantial economic losses [7,8,9,10]. The 2020 desert locust crisis in East Africa serves as a dramatic example, where massive swarms invaded millions of hectares of farmland, directly endangering the livelihoods and food supplies of millions [1,11,12].

However, the impact of locust outbreaks reaches far beyond immediate agricultural damage. Their feeding activity not only drastically reduces pasture and crop yields but also leads to large-scale degradation of plant communities. This, in turn, exacerbates soil erosion and water loss, ultimately undermining the stability and resilience of entire ecosystems [13,14,15]. In vulnerable regions like grasslands and agro-pastoral transition zones, grasshoppers occur more commonly. While their outbreaks are generally smaller in scale, they similarly diminish the ability of grassland vegetation to sequester carbon and retain soil moisture, leading to a reduction in ecosystem services. This decline creates significant challenges and escalates the costs of ecological restoration [16,17]. Although both locusts and grasshoppers belong to the same family (Orthoptera: Acrididae), their outbreaks differ substantially in intensity and scale [18]. Locusts tend to have a stronger aggregative behavior, allowing them to form dense swarms that cause extensive damage [6]. In contrast, grasshopper outbreaks are generally more localized, with smaller group sizes and less intense migration patterns [19]. Nevertheless, the early warning systems for locusts and grasshoppers outbreaks are equally crucial. The technical focus, however, may differ: for locusts, the emphasis is often on monitoring cross-regional migration patterns and responding to large-scale spread, while for grasshoppers, the focus is more on early detection and localized high-density outbreaks [10].

### 1.2. The Spatiotemporal Complexity of Locust/Grasshopper Population Dynamics

Accurately predicting locust outbreaks is a formidable challenge due to the inherent complexity of the process in both space and time. Locust population development, migration, and outbreak are driven by a combination of environmental factors, including weather conditions (e.g., temperature, precipitation, and extreme events), vegetation characteristics (e.g., type, coverage, and phenology), topography, and human activities [20,21]. These factors interact in complex, nonlinear ways, and their influence is not uniform across locations and times. For instance, identical rainfall amounts might result in high egg hatching success in one area due to favorable vegetation cover but have little effect in another area with different soil conditions [22,23]. Furthermore, local population dynamics are seldom isolated. Source populations in one region can trigger outbreaks in another through migration, creating long-distance ecological linkages [24,25]. This migration mobility often causes strong spatial autocorrelation, as locusts typically move in large groups over vast distances, with outbreaks in one region increasing the likelihood of outbreaks in nearby areas, thereby enhancing the risk of cross-regional ecological chain transmission [26]. In contrast, grasshopper migration tends to exhibit weaker spatial autocorrelation. Their group sizes and migration ranges are smaller, and they are more strongly influenced by local environmental conditions. While grasshoppers may form high-density populations in specific areas under suitable climatic conditions, their spread is usually constrained by local ecological factors and resource availability, lacking the extensive cross-regional migration seen in locusts [19,27]. Given the multifaceted nature of locust and grasshopper outbreaks, as well as the spatial correlations at different scales, effective predictive modeling requires the ability to handle heterogeneous data, capture nonlinear relationships, and interpret both spatial and temporal dynamics simultaneously. This challenge has driven ongoing innovation in predictive modeling methods within this field.

### 1.3. Critical Environmental Drivers for Locusts/Grasshoppers

The population dynamics of locusts are highly sensitive to meteorological conditions, with temperature and precipitation being particularly influential [28,29,30]. Favorable thermal conditions accelerate development and enhance reproductive output, while moderate rainfall provides essential soil moisture for egg hatching and nymph survival [31]. However, this dependency also implies vulnerability; extreme weather events, such as intense precipitation, can sharply reduce populations by causing egg mortality, thereby disrupting the life cycle [32]. Notably, locust reproductive peaks often coincide with the warm, humid conditions of summer, which also aligns with crucial stages of plant growth [33,34]. This synchrony between locust life history and host plant phenology adds considerable complexity to forecasting efforts.

Beyond weather, vegetation cover acts as another critical and multi-faceted driver. It directly affects locust survival across various life stages, serving as a food source, providing habitat for shelter and oviposition, and even influencing migratory behavior [33,35]. Dense vegetation offers ample forage, which can support population growth [34,36]. Moreover, it creates suitable microhabitats that help regulate soil moisture and temperature, establishing conditions that promote egg development and nymph survival [37]. Therefore, an effective predictive framework for locust population dynamics must integrate both meteorological and vegetation data. Such an integrated approach is essential for improving outbreak forecasting, unraveling the ecological mechanisms behind population fluctuations, and ultimately providing a scientific basis for targeted pest control strategies.

In contrast, while grasshopper population dynamics are also influenced by temperature, precipitation, and vegetation cover, their responses are generally more localized. Grasshoppers typically form high-density populations under suitable climatic and vegetation conditions, but their growth and spread are largely constrained by local habitat characteristics, resource availability, and microclimatic conditions [19,26,27]. Unlike locusts, grasshoppers do not exhibit the large-scale, long-distance migration patterns.

## 2. Scope, Objectives, and Structure of This Review

Locust outbreak patterns and their primary environmental drivers vary significantly across the world and are influenced by distinct geographic and ecological settings [38]. These regional differences are critical. For example, in Inner Mongolia’s grassland ecosystem, outbreaks display clear regional and periodic characteristics. They occur primarily in typical grasslands, desert grasslands, and agro-pastoral transition zones, each harboring its own dominant locust species and responding to unique environmental drivers [28,29]. This review provides a comparative analysis of different modeling paradigms, with a central focus on their application to the critical issue of predicting locust outbreaks. While the discussion covers traditional statistical methods, classical machine learning, and deep learning techniques, the scientific motivation, evaluation benchmarks, and practical utility are closely tied to the ecological and operational complexities of locust forecasting. The review uses locust outbreaks as a key example to demonstrate how advanced modeling approaches, particularly deep learning, can be applied to address complex nonlinear systems in ecological forecasting.

To begin, we outline the ecological foundation by examining the key factors influencing locust population dynamics and regional outbreak patterns. Subsequently, we trace the methodological progression from traditional statistics to machine learning and, ultimately, to deep learning, offering a critical appraisal of the current state, strengths, and weaknesses of various DL architectures. Building on this analysis and taking into account the pest management needs of Inner Mongolia, we focus on the Gated Recurrent Unit (GRU), exploring its specific potential for locust time-series prediction and proposing future directions, including model interpretability and cross-regional generalization.

Through this structure, our goal is to provide the research community with a clear roadmap of technological progress and future insights. We seek to promote the theoretical and practical adoption of deep learning in locust monitoring. A central theme of this work is to highlight a significant research gap: despite the transformative potential of DL, its application remains limited for non-desert locusts, especially in data-scarce ecosystems like Inner Mongolia’s grasslands. Addressing this gap is a task of considerable scientific and practical urgency. To clarify the conceptual foundation of this review, a brief note on terminology is necessary. The term “locust” is widely used in the literature, reflecting a historical and geographical bias toward highly migratory species in arid regions, such as the desert locust. However, the species responsible for outbreaks in Inner Mongolia’s grasslands are more accurately classified as “grasshoppers” in a strict taxonomic sense—a term commonly used in regional studies. Despite this, these grasshoppers exhibit population explosion and collective behavior that are functionally analogous to locust plagues, effectively making them “grasshopper locusts” in an ecological and economic context. Therefore, while this review uses the widely recognized term “locust” to maintain consistency with the global methodological discourse, its core mission is to bridge this divide. We focus specifically on adapting predictive insights—largely gleaned from desert locust research—to the unique challenges of forecasting grasshopper outbreaks in vital grassland ecosystems.

## 3. The Evolutionary Pathway of Classical Modeling: From Statistics to Machine Learning

The pursuit of predicting locust outbreaks has followed a clear path of methodological advancement, beginning with classical statistical techniques and evolving toward machine learning. These foundational approaches provided the initial computational strategies for understanding the complex factors driving locust populations, laying the groundwork for modern early warning systems. Tracing this progression shows how these early methods were essential stepping stones to the contemporary deep learning paradigms discussed later.

### 3.1. Traditional Statistical Models

Traditional statistical models were pioneers in locust prediction, establishing key theoretical and practical foundations for disaster prevention. These methods generally fall into three categories: regression models, time-series models and Bayesian models.

Regression modeling seeks to create predictive frameworks by mathematically quantifying the relationship between locust occurrence and environmental drivers like temperature and precipitation [39]. One common technique, multiple linear regression, can reveal how favorable thermal conditions and rainfall promote population growth while also capturing declines linked to extreme weather events [39,40].

In contrast, time-series models focus on extracting patterns from historical pest data to forecast future trends. The Autoregressive Integrated Moving Average (ARIMA) model, a classic example, combines autoregressive, differencing, and moving average components to model underlying trends, seasonality, and random noise, proving effective for short-term locust intensity forecasting [30]. To better account for the seasonal nature of outbreaks, the Seasonal ARIMA (SARIMA) model incorporates seasonal components, improving the ability to capture fixed-period dynamics [41].

Bayesian statistical frameworks further extend traditional approaches by explicitly modeling temporal dynamics and uncertainty while also allowing sequential updates as new data become available [42]. Using hierarchical or state-space formulations, Bayesian models have been successfully applied to locust forecasting, particularly in data-limited or non-stationary conditions [43,44]. These methods integrate prior ecological knowledge with observational data, offering an interpretable and flexible framework for understanding population dynamics over time [25].

Although traditional statistical models work well in stable data environments, they struggle to capture the complex spatiotemporal dynamics of locust populations. The assumptions of linearity and stability limit their ability to model the nonlinear relationships between environmental factors and locust behavior [21,43]. Extended methods, such as Bayesian models, can handle time dependence, but they typically require extensive model settings and computational resources, which limits their scalability and operational efficiency [45]. Despite their strong theoretical advantages in modeling uncertainty and temporal evolution, Bayesian methods face challenges in large-scale spatiotemporal predictions, especially in integrating spatial and long-range temporal dependencies, such as locust migration [46,47]. These limitations have driven the search for more advanced methods, opening the door to machine learning and deep learning approaches.

### 3.2. Machine Learning

To overcome the limitations of traditional statistical models, the field saw a significant shift with the introduction of machine learning (ML). ML algorithms, such as Random Forest (RF), Support Vector Machines (SVMs), and Maximum Entropy (MaxEnt), excel at uncovering complex, nonlinear relationships within multi-source environmental data, leading to substantial improvement in predictive accuracy.

Random Forest (RF), a robust ensemble method, is particularly effective in feature selection. By leveraging multiple decision trees, RF evaluates a range of predictors—such as vegetation indices, soil moisture, and precipitation patterns—to identify subtle environmental triggers that favor locust outbreaks [48,49]. Its integration with remote sensing data has proven highly reliable for estimating locust density [50]. Furthermore, RF’s iterative sampling strategy helps mitigate overfitting and contributes to its strong generalization capability, as validated in several studies [51].

Similarly, Support Vector Machines (SVMs) have proven effective in both classification and regression tasks. The algorithm constructs hyperplanes that maximize the classification margin in high-dimensional space, enabling it to distinguish outbreak from non-outbreak conditions. Its kernel functions are especially useful for capturing the complex nonlinear patterns between environmental conditions and locust dynamics [52,53]. Research suggests that hybrid approaches combining RF and SVMs can enhance performance, especially when processing feature-rich and noisy datasets, providing more robust support for early warning systems [54,55]. Beyond these models, broader ML techniques like dimensionality reduction, data augmentation, and transfer learning provide valuable tools for enhancing model scalability, managing high-dimensional data, and improving generalization in data-sparse regions [56].

Another prominent ML approach in locust prediction is the Maximum Entropy (MaxEnt) algorithm, which is widely used in species distribution modeling and habitat suitability analysis [57,58]. MaxEnt’s core strength lies in its ability to model a species’ probability distribution across a landscape based on environmental variables, following the principle of maximum entropy [59]. A major advantage of MaxEnt is its reliability with sparse or incomplete data, a common scenario in ecology. It performs well with limited training samples and can effectively handle variables like temperature, precipitation, and vegetation type [60]. In locust prediction, MaxEnt has been extensively employed to model potential habitats and forecast regions where locusts are likely to thrive based on their ecological niche [25,26,61,62]. Moreover, MaxEnt’s outputs are highly interpretable, generating habitat suitability maps that can guide targeted pest control intervention [63].

Despite their contributions, these classical machine learning methods share fundamental limitations in modeling the spatiotemporal complexity of locust outbreaks. For example, RF typically treats data points as independent observations and lacks native mechanisms to model temporal dependencies, unless temporal features are explicitly engineered or lagged variables are manually incorporated. This limits its effectiveness in capturing dynamic processes such as migration and population growth [64]. The SVM faces scalability challenges, as its computational cost can become prohibitive with large-scale spatial data or complex kernels, hindering its use in real-time forecasting [65]. Both RF and the SVM are also highly sensitive to data quality, with performance degrading significantly in the presence of noise, missing values, or inconsistencies [66]. While MaxEnt excels at identifying spatially suitable habitats, it is inherently a static model. Temporal dynamics can only be indirectly explored by applying MaxEnt to time-specific environmental layers or projected future conditions, which requires pre-modeling the temporal evolution of predictors. This method does not allow for the direct learning of temporal dependencies within the model itself [67,68,69].

Although integrating these models can sometimes provide complementary benefits, such combinations often amplify their individual weaknesses. A particular challenge is the difficulty in fusing MaxEnt’s spatial outputs with the limited temporal capabilities of RF or SVMs, resulting in computationally intensive frameworks that still fail to capture the spatiotemporal drivers of locust outbreaks [70,71]. These limitations—specifically the inadequate handling of spatiotemporal components and reliance on manual feature engineering and data quality—highlight the need for a more powerful approach. This unmet demand paved the way for deep learning, which offers groundbreaking potential by automatically learning features and directly processing complex spatiotemporal dependencies. 

## 4. Deep Learning Architectures for Locust Prediction

In recent years, deep learning has significant advanced locust forecasting (the comparison of these 3 types of modelling models shows in Table 1). Unlike conventional machine learning, deep learning excels at capturing complex nonlinear and spatiotemporal relationships directly from data. Its key advantage lies in its ability to extract features adaptively, thereby reducing the reliance on manual feature engineering. By modeling interactions within multi-source, heterogeneous datasets, deep learning enhances prediction accuracy and robustness, overcoming the limitations of traditional methods and enabling more effective monitoring and early warning systems.

### 4.1. Deep Neural Networks (DNNs): Basic Framework and Multi-Dimensional Environment Modeling

As an early and foundational deep learning architecture, Deep Neural Networks (DNNs) have shown considerable promise in predictive modeling. While their direct applications to locust prediction are still developing, their proven ability to model dynamic processes and recognize environmental patterns makes them highly applicable to this task. DNNs are well-suited to analyze multi-dimensional environmental data—such as climate variables, habitat features, and soil properties—to forecast locust migration paths, reproductive cycles, and habitat suitability [71,72,73,74].

One of the key strengths of DNNs in ecological contexts is their capacity to learn reliable patterns from high-dimensional datasets, even when field samples are limited [73]. Additionally, their neural architecture can simulate interactions between organisms and their environment, enabling the integration of satellite and ground-based data to predict migration patterns and key life-cycle events [75]. However, these capabilities come with trade-offs. DNNs are less effective at capturing local or hierarchical spatial structures within data. They tend to underperform when applied to highly structured, high-dimensional data like images, making them better suited for analyzing simpler, unstructured datasets or processed feature vectors [76,77,78].

### 4.2. Convolutional Neural Networks (CNNs): Excelling in Spatial Feature Extraction

Convolutional Neural Networks (CNNs) are particularly effective for spatial feature extraction, a capability derived from their convolutional and pooling layers. These layers allow the network to retain crucial spatial information while significantly reducing the number of parameters, making the model more efficient [79,80,81]. The core advantage of this architecture lies in two key mechanisms: local receptive fields and weight sharing. These features enable CNNs to efficiently detect local patterns and complex spatial dependencies across various data types, including remote sensing imagery [82,83], topographic maps [84], and other gridded spatial data [85].

In locust prediction, this spatial process is directly applied to multi-band remote sensing data from sources like Sentinel-2 and Landsat. CNNs can automatically extract features related to locust habitat quality and breeding potential, such as vegetation indices (e.g., NDVI), land surface temperature, and soil moisture. A notable example of this capability is the work by Samil et al. [86], who used CNNs to model vegetation greenness and soil moisture in East Africa, successfully identifying potential breeding hotspots for desert locusts in complex environmental settings.

Looking forward, a prominent research trend is integrating CNNs with attention mechanisms to enhance prediction accuracy. This combination allows models to focus on the most relevant regions within an image—those providing the strongest signals of an impending outbreak. This approach not only improves feature relevance and prediction robustness but also enhances the interpretability of the model’s decisions [75,87,88].

### 4.3. Recurrent Neural Networks (RNNs) and LSTM: Mastering Temporal Dependencies

Recurrent Neural Networks (RNNs) represent a specialized class of architectures designed for sequential data. Their internal feedback structure grants them memory, making them suitable for capturing short-term temporal dependencies and forecasting dynamic changes [89]. A significant limitation of traditional RNNs, however, is their difficulty in learning long-range dependencies due to issues like vanishing or exploding gradients during training [90].

The Long Short-Term Memory (LSTM) network was introduced to overcome this limitation. LSTM networks address this with a gated structure that includes input, forget, and output gates. This design allows the network to effectively regulate information flow, selectively retaining important long-range information while discarding irrelevant details, thereby dramatically improving the modeling of long-term temporal dependencies [91,92].

In locust prediction, the ability to model temporal dynamics is crucial. LSTM networks can effectively integrate historical locust data with the time series of meteorological observations (e.g., temperature and precipitation) and vegetation phenology indicators (e.g., NDVI) to simulate how populations dynamically respond to environmental changes. For example, Samil et al. [86] demonstrated that an LSTM network could forecast regional locust swarm distributions a month in advance with high spatial accuracy. This approach has been further refined in subsequent work, including the LocustLens data fusion system [93] and frameworks designed for long-term swarm dynamics [94]. These applications highlight that LSTM networks offer a robust foundation for capturing the complex, nonlinear, and often delayed environmental feedbacks that characterize locust ecology.

Current research continues to expand these capabilities, exploring the integration of bidirectional LSTM networks with attention mechanisms. This allows models to capture more complex temporal patterns by considering both past and future contexts, as well as automatically weighting the importance of key time points. These advances further enhance both prediction accuracy and model interpretability [95,96], contributing to the development of more reliable locust early warning systems.

### 4.4. Hybrid Models: The CNN-LSTM Paradigm for Integrated Spatiotemporal Analysis

Locust outbreaks occur across both space and time, making the development of hybrid models that combine Convolutional and Recurrent Neural Networks (CNN-LSTM) a key innovation. These models integrate the spatial feature extraction process of CNNs with the temporal dynamic modeling strengths of LSTM networks, resulting in superior performance in locust forecasting.

The typical CNN-LSTM framework follows a two-stage process: A front-end CNN processes remote sensing imagery to extract key spatial features like vegetation distribution and soil moisture. These features are then passed to a back-end LSTM network that models their temporal evolution. This creates an integrated “spatial recognition-temporal prediction” pipeline [97,98].

This hybrid approach has proven highly effective in practice. For instance, Yusuf et al. [99] used an LSTM-based recurrent convolutional network to capture spatiotemporal dynamics between climate variables and locust reproduction, utilizing data from the FAO. In another significant study, Shao et al. [75] used CNNs to extract vegetation coverage from MODIS imagery and LSTM networks to predict locust density 1–3 months ahead, demonstrating significantly higher accuracy than standalone models. The versatility of this architecture is further evidenced by its successful application in other spatiotemporal forecasting domains, such as air pollution monitoring [100,101], highlighting its broad applicability. An advanced iteration of this model, the Attention-based LSTM Interaction CNN (ALIC) model, has shown even greater accuracy and interpretability in linking meteorological variables to pest dynamics [102].

However, the enhanced capability of CNN-LSTM models comes with practical trade-offs. Their architectural complexity demands substantial computational resources and large volumes of high-quality labeled data for training—a particular challenge in data-scarce environments like those for grassland locust monitoring [103]. Additionally, the extensive parameter set requires careful tuning, complicating deployment in operational systems [104,105]. Moreover, as the input sequence length grows, the computational burden of the LSTM component increases significantly, potentially hindering real-time forecasting [106,107]. In response to these challenges, current research is focusing on more efficient solutions, including lightweight architectures, hybrid attention mechanisms, and adaptive parameter-sharing networks.

### 4.5. The Gated Recurrent Unit (GRU): An Efficient and Potent Alternative

As a more streamlined alternative to the LSTM approach, the Gated Recurrent Unit (GRU) offers an appealing balance between performance and efficiency. Introduced by Cho et al. [108], the GRU simplifies the LSTM architecture by combining the input and forget gates into a single update gate while retaining a reset gate to control information flow [108,109]. This elegant design preserves the ability to model long- and short-term dependencies but reduces the number of trainable parameters, leading to faster training times and a smaller computational footprint while effectively addressing the vanishing gradient problem [110].

These characteristics make the GRU particularly advantageous in resource-constrained or time-sensitive scenarios. Its efficiency has been demonstrated across various fields requiring robust temporal modeling, including hydrological forecasting [111], intelligent transportation systems [112], and industrial equipment monitoring [113]. Notably, GRUs have also shown a remarkable ability to perform well with small sample sizes, learning effective patterns rapidly and resisting overfitting—a significant advantage in data-limited context [114]. This makes them exceptionally well-suited for meteorological time-series forecasting, where they have been successfully applied to predict variables like temperature, humidity, and precipitation [115,116].

Although the application of GRUs to locust population prediction is still emerging, their proven success in modeling meteorological and other ecological time-series data strongly suggests their potential [117,118]. Given the dependence of locust life cycles on the climate, the GRU’s computational efficiency, adaptability to small datasets, and proven ability in weather-driven modeling position it as a highly promising tool. It offers the potential to efficiently model the nonlinear relationships between key meteorological drivers and locust population dynamics, providing a viable approach for predicting both population fluctuations and spatiotemporal dispersal patterns, especially in regions like Inner Mongolia where historical monitoring data may be limited.

In conclusion, the current research and application status of 5 major deep learning models in locust prediction is summarized in a tabular form (Table 2), providing a reference for the rational selection of model types.

## 5. Challenges, Research Gaps, and Future Directions

### 5.1. Predominant Challenges in Current DL-Based Prediction

While deep learning (DL) holds significant potential for revolutionizing locust prediction, its transition from a promising tool to a reliable solution in the field faces several interconnected challenges. A clear and systematic approach to addressing these obstacles is essential for unlocking the full potential of DL for proactive pest management.

#### 5.1.1. The Data Bottleneck: Scarcity, Heterogeneity, and Quality

The effectiveness of deep learning models is fundamentally constrained by the availability and quality of training data [119], and locust monitoring faces a significant data bottleneck. A primary issue is sheer data scarcity. Collecting high-quality, field-validated locust data (e.g., population density) is both costly and logistically difficult, particularly in expansive ecosystems like the Inner Mongolian grasslands. This frequently results in sample sizes that are too small to support robust model training [120].

Compounding the problem of scarcity is the profound heterogeneity of the multi-source data required for prediction. Satellite imagery, meteorological reanalysis, and ground observations often differ in their temporal and spatial resolution, data formats, and collection standards, creating a significant technical challenge in building unified, coherent datasets [121].

Moreover, data quality is a critical concern. Imperfections—such as sensor errors, communication failures, and inconsistencies in human recording—frequently introduce noise and missing values into the datasets [122]. Deep learning models are highly sensitive to these flaws, which can severely affect the performance and real-world reliability of even the most sophisticated prediction systems [123,124].

#### 5.1.2. The Generalization Gap and the Neglect of Grassland Ecosystems

A key limitation of deep learning is its constrained generalization capability. Models that are carefully tuned to data from one region or locust species often fail when applied to new contexts, as they tend to learn dataset-specific superficial patterns rather than underlying universal ecological principles [50,71].

This generalization gap is further compounded by a pronounced research bias, an overemphasis on the desert locust (*Schistocerca gregaria*), which has dominated the literature [61,75,99,125]. The environmental drivers, population dynamics, and management strategies for desert locusts differ fundamentally from those of the species endemic to grassland ecosystems. As a result, models developed for arid desert environments often experience significant performance degradation when applied to grassland contexts. This disparity highlights a critical research gap and underscores the urgent need for modeling approaches specifically designed for these ecologically and economically important, yet understudied, regions [126,127].

#### 5.1.3. The “Black Box” Problem and the Need for Explainability

While deep learning models possess remarkable predictive power, they are often plagued by a significant drawback: a lack of model interpretability, commonly known as the “black box” problem. Despite achieving high accuracy, these models obscure the decision-making process, making it difficult to identify which environmental drivers are influencing predictions or to understand the reasoning behind specific forecasts [128].

In practical pest management, this lack of transparency presents a major barrier to adoption. Beyond simply knowing when and where an outbreak might occur, managers and policymakers need to understand why a model makes a particular prediction. This explanatory layer is essential for designing targeted and ecologically appropriate interventions. For instance, understanding whether a predicted outbreak is primarily driven by vegetation density or soil moisture is critical for deciding whether to focus resources on habitat management or water source control [129].

Ultimately, without credible explanations for their predictions, even the most accurate models struggle to gain the trust of end-users, severely limiting their practical utility and hindering integration into real-world decision-making processes.

### 5.2. Promising Avenues for Future Research

Addressing the challenges outlined above requires a shift towards deep learning paradigms that are more data-efficient, interpretable, and grounded in ecological principles. A promising path forward integrates several advanced technological strategies, with a core focus on maximizing the utility of limited data and enhancing model generalization [130].

To directly address the data bottleneck, future work should leverage transfer learning to adapt knowledge from data-rich domains and mitigate scarcity in target regions [131,132]. This approach can be combined with advanced data augmentation techniques, particularly Generative Adversarial Networks (GANs), which hold unique promise for generating realistic locust occurrence scenarios across diverse environmental conditions, thereby artificially expanding training sets and bolstering model robustness [133].

However, overcoming current limitations requires more than a sole focus on predictive accuracy. A parallel and equally critical endeavor is to embed ecological realism and transparency into the models. The integration of Explainable AI (XAI) tools, such as SHAP and LIME, can illuminate the “black box” decision-making process, identify key environmental drivers, and build the trust necessary for operational adoption [134,135]. In addition, incorporating mechanistic microclimate models, such as NicheMapR [136,137], offers a promising avenue to derive biologically meaningful predictors (e.g., body temperature, thermal stress, and water balance), which are particularly relevant for locust forecasting in grassland ecosystems and can substantially enhance ecological interpretability. Furthermore, Physics-Informed Neural Networks (PINNs) offer a groundbreaking avenue for incorporating established ecological principles—for instance, insect developmental rate equations—directly into the learning process. This guides models toward biologically plausible solutions, enhancing both interpretability and generalization capability [138].

The true potential lies in merging these approaches to construct end-to-end predictive frameworks for critical, yet understudied, ecosystems. A prime exemplar is the development of a GRU-based prediction framework for grasshopper locusts in grassland ecosystems. The GRU’s efficiency and effectiveness with temporal data make it well-suited for data-scarce settings and for modeling the key meteorological time series that drive locust population dynamics [115,116,117,118,139,140]. By combining multi-source data, transfer learning, GAN-based augmentation, and insights from XAI or PINNs, such a framework would not only provide accurate forecasts but also serve as a platform for scientific discovery, helping to unravel the underlying ecological mechanisms of outbreaks. It would transform predictive models from opaque tools into transparent engines for actionable decision support.

**Figure 1:**
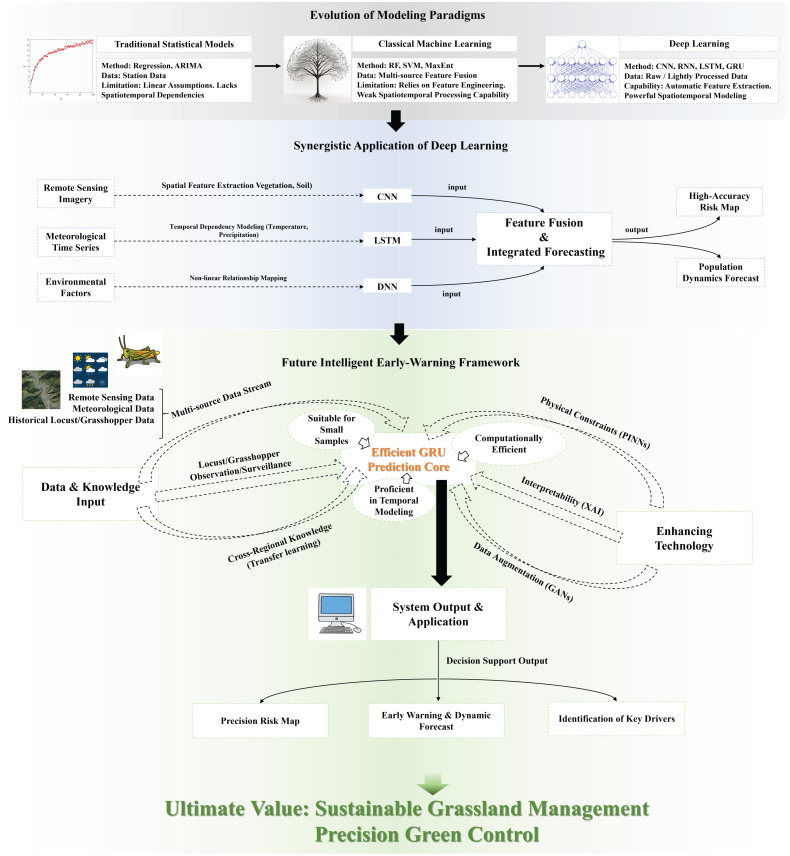
Evolution of locust prediction technology and a future intelligent early warning framework for grassland ecosystems.

This conceptual diagram illustrates a three-part progression in locust forecasting (Figure 1). The first part (gray) outlines the paradigm shift from traditional statistical models (reliant on linear assumptions and station data) to classical machine learning (handling nonlinearity with engineered features) and finally to deep learning (enabling automatic feature extraction from raw, complex data). The second part (blue) demonstrates the synergistic application of core deep learning architectures, where Convolutional Neural Networks (CNNs) extract spatial features from remote sensing imagery, Long Short-Term Memory (LSTM) networks model temporal dependencies in meteorological sequences, and Deep Neural Networks (DNNs) capture nonlinear relationships with environmental factors; their outputs are integrated for spatiotemporal forecasting. The third part (green) proposes a future intelligent framework tailored for data-scarce grassland ecosystems. Centered on an efficient Gated Recurrent Unit (GRU) prediction core, this framework is enhanced by key enabling technologies: transfer learning to adapt knowledge from data-rich regions, Generative Adversarial Networks (GANs) for data augmentation, Physics-Informed Neural Networks (PINNs) to integrate ecological principles, and Explainable AI (XAI) to clarify key drivers behind predictions. This integrated system is designed to overcome challenges of data scarcity, model generalization, and interpretability, ultimately generating actionable and ecologically grounded intelligence for sustainable pest management in vulnerable regions.

## 6. Conclusions

The spatiotemporally complex and nonlinear nature of locust ecology has driven the evolution of prediction methodologies, from statistical models to deep learning architectures capable of deciphering these patterns. While deep learning holds transformative potential, its practical application is constrained by challenges such as data scarcity, limited generalization, and interpretability issues. Therefore, the way forward should not focus solely on developing models for the sake of modeling but on an ecologically grounded strategy. It includes integrating transfer learning and generative models to overcome data limitations. It also involves employing XAI and physics-informed learning to reveal model decision-making and embed ecological realism. Finally, applying tailored approaches, with efficient architectures like GRUs at their core, towards critical yet underserved ecosystems (like grasslands) is important. The ultimate goal is to shift from simply generating forecasts to building transparent, actionable decision-support systems that are as dynamic as the pest systems they aim to manage, thereby effectively bridging computational power with the on-the-ground demands of sustainable ecosystem stewardship.

## Figures and Tables

**Table 1:** Comparison of ecological forecasting modeling methods: integrating statistical models, machine learning, and deep learning.

Comparison Aspect	Traditional Statistical Models	Classical Machine Learning	Deep Learning (Overall)
Core Principle	Parametric forms; linear or pre-specified nonlinear assumptions	Learns nonlinear patterns from engineered features	End-to-end learning; automatic feature extraction from raw data
Representative Algorithms	Multiple linear regression, ARIMA, SARIMA, and Bayesian	RF, SVMs, and MaxEnt	Neural Networks (e.g., CNN, RNN, LSTM, and GRU)
Handled Data	Structured tabular data	Structured tabular data; multi-source feature	Complex raw data (images, time series, and spatial grids)
Nonlinearity Modeling	Limited to predefined functional forms	Core strength; captures complex interactions	Core strength; universal approximator for hierarchical patterns
Temporal Dynamics Modeling	Explicit but requires stationarity assumptions and manual specification (e.g., ARIMA). Bayesian models allow sequential updating	Not inherent; relies on feature engineering	Inherent; architectures (e.g., LSTM/GRU) learn dependencies automatically
Key Advantages	High interpretability, simple, and low cost	Feature importance/suitability maps; handles complex nonlinearities	Powerful spatiotemporal modeling, highest predictive potential, automatic feature learning
Main Limitations (for Locust Prediction)	Struggles with high-dimensional, complex interactions; scalability issues	Heavy reliance on feature engineering; poor native spatiotemporal integration	“Black box”, high data demand, high computational cost, and poor generalization if not careful
Ecological Interpretation	High	Medium (e.g., MaxEnt provides suitability maps)	Low (requires XAI techniques)
Application Scenarios	Preliminary analysis/short-term forecasting; uncertainty-focused risk assessment	Static habitat mapping (MaxEnt); driver identification; medium-term warning	Large-scale, multi-source, and complex spatiotemporal dynamic forecasting

**Table 2:** Comparison of deep learning architectures applicable to locust prediction.

Comparison Aspect	DNN	CNN	RNN/LSTM	CNN-LSTM	GRU
Core Architectural Traits	Multiple fully connected (dense) layers.	Convolutional layers and pooling layers; local connectivity and weight sharing.	Recurrent connections with internal state (LSTM includes gating mechanisms).	CNN front-end + LSTM back-end in sequence.	Variant of LSTM; simplified gating (update gate and reset gate).
Primary Data Type Handled	Tabular data; feature vectors.	Gridded spatial data (e.g., remote sensing imagery).	Time-series data.	Spatiotemporal sequence data (e.g., multi-temporal remote sensing imagery).	Time-series data.
Key Strengths	Powerful nonlinear fitting capability; foundational framework.	Excellent spatial feature extraction and translation invariance.	Excellent temporal dependency modeling (LSTM handles long-range dependencies).	Captures both spatial features and temporal evolution; strong comprehensive capability.	Performance comparable to LSTM but simpler structure, faster training, and more robust with small samples.
Typical Application in Locust Prediction	Mapping environmental factors to occurrence risk.	Identifying potential breeding areas from remote sensing images.	Predicting population changes driven by meteorological sequences.	Predicting spatiotemporal spread and outbreak trends of locusts.	(High potential) Efficiently modeling climate-driven population dynamics.
Main Limitations	Ignores spatial structure of data; unsuitable for images or sequences directly.	Difficulty handling temporal dynamics directly.	Difficulty handling spatial information directly; the RNN suffers from vanishing gradients.	Complex model, high training cost, and large number of parameters.	Emerging model; lacks extensive application examples in locust prediction specifically.
Representative Studies	[73]	[86]	[86,94]	[75,99]	Proposed based on [115,117]

## Data Availability

No new data were created or analyzed in this study. Data sharing is not applicable to this article.
